# Neo-Naturalism, Conciliatory Explanations, and Spatiotemporal Surprises

**DOI:** 10.3389/fpsyg.2018.02506

**Published:** 2019-01-14

**Authors:** Uziel Awret

**Affiliations:** Department of Physics, Inspire Institute, Falls Church, VA, United States

**Keywords:** consciousness, naturalism, meta-problem, ignorance hypothesis, emergent spacetime, holographic principle, type-C materialism

## Abstract

Some materialists believe that physics is rich enough to bridge Levine's Explanatory Gap^1^, while others believe that it is not. Here I promote an intermediate position holding that physics is rich enough to explain why this gap seems more intractable than similar inter-theoretic explanatory gaps, without providing a full-blown “physical” explanation of consciousness. At a minimum, such an approach needs to explore the prospects of empirical discoveries that can diminish the power of anti-physicalist arguments like Chalmers's “conceivability argument”^2^ and Jackson's “knowledge argument.” While this is not an easy task, recent advances in the physics of spacetime and information convince us that these prospects are not poor. The empirical bent of this approach suggests framing it as a naturalist theory of mind seeking to situate or make room for consciousness within our great naturalist system, but the reliance of this approach on recent (re)conceptions of time and information pulls the carpet out from under essential concepts like concreteness and causation, thus demanding a radically reconfigured naturalism, or *neo-naturalism*. The question that will frame this discussion is, “What could possibly count as an empirical fact that can help naturalize consciousness?”

## Introduction

In the first five sections, I seek to present the ingredients of the aforementioned approach, while in the sixth section, I will apply this approach to three specific examples.

Sec. 1. Naturalism and Neo-Naturalism.
  1a. Naturalism.
  1b. Setting the limits from within 1c. Neo-Naturalism.
Sec. 2. Chalmers's A–F classification of theories of mind and the meta-problem of consciousness.
  2a. The meta-problem.
  2b. The six theories.
Sec. 3. Two problems facing naturalized type-C theories, SDA, Stoljar's Ignorance Hypothesis, and Transformational Emergence.
  3a. Naturalism and the Ignorance Hypothesis.
  3b. Transformational Emergence.
  3c. The SDA and type-XC theories.
4. Unification, poiesis, and symmetry.
  4a. Unificationist Neo-Naturalism.
  4b. Revelatory dynamics.
  4c. Noether's Theorem.
Sec. 5. Neo-naturalizing information and Pragmatic Neutral Monism.
  5a. Common Origin Pragmatic-Neutral Monism.
  5b. Neo-naturalizing information.
Sec. 6. Three physical examples that can make a philosophical difference.
  6a. Self-measurement, circumventing the SDA.
  6b. Chronos and Kiros.
  6c. Holographic possibility.

## Sec. 1. Naturalism and Neo-Naturalism

### 1a. Naturalism

Unlike physicalism, naturalism is not a thesis but a loose collection of ontological and methodological commitments that might best be described as a cherished “coat of arms” portraying a scientist slaying a supernatural dragon. At the same time, here is Melnyk (2013) on the difference between philosophy today and 30 years ago:

The obvious outward sign of this difference in practice is the greatly increased probability that a philosophical journal article or book will discuss or cite the findings of some kind of empirical investigation, usually a science, but sometimes a branch of history. The difference itself is the (partial) so-called naturalization of many branches of philosophy (p. 79).

Born out of opposition to claims of the supernatural, naturalism today is mostly associated with a rejection of a priori analytic attempts to restrict the scope of the sciences and a strict adherence to the causal closure of the physical, rejecting realist approaches to mathematical and normative concepts and epiphenomenal approaches to consciousness. Ontological naturalism rejects spatiotemporal effects that lack spatiotemporal causes (Papineau, 2016). Naturalism comes in many stripes and Neo-Naturalism even more so, as, like the guests in Sodom and Gomorrah, naturalism can be stretched Chalmers's “naturalistic dualism” (Chalmers, 1996) or shrunk (Dennett's eliminativist naturalism) to fit most theories of mind. While a conservative view of reality defined in opposition to the supernatural, naturalism seems to respect Pre-Socratic intuitions of physics as a poietic unfolding and a source of novel revelation. Naturalism has a special relation with the sciences, and despite favoring the empirical, synthetic, and a posteriori, is not opposed to a priori analysis as long as it does not conflict with the empirical program (Quine, 1969) of “Naturalizing Epistemology.”

The inevitable march of physics toward background-independent physical theories^3^ and emergent spacetime threatens traditional ontological naturalism with an unparalleled crisis, bringing into conflict its two bedrock commitments, the one to the sciences and the other to spatiotemporal causal closure. In the spirit of “never let a good crisis go to waste,” I will treat this crisis as an opportunity to formulate a neo-naturalist theory of mind, relying on novel approaches to spacetime and providing a more nuanced ontology. To quote Humphreys (2016a) in *New directions in philosophy of science*:

The realm of the non-mental, which is how the domain of physicalism is usually construed, contains a remarkable variety of ontological and theoretical features, and that variety must be respected rather than lumped into the catchall category of physicalism (p. 6).

According to Papineau (p. 6), the driving motivation for ontological naturalism is the need to explain how different kinds of things can make a causal difference to the spatiotemporal world. Ontological naturalism's unconditional embrace of spatiotemporal causal closure leaves it with two unsatisfactory choices—either consciousness unfolds in space and time like ordinary physical entities, which entails all the problems faced by physicalism, or else it is somehow “external” to space and time, facing the problems entailed by dualism [see Papineau on Interactionist Dualism; (Papineau, 2016), p. 16 (A4)]. However, the aforementioned crisis presents us with a third possibility that harbors genuinely novel moves that can help “make room” for consciousness within our great naturalistic system (without fully explaining it) by providing empirical reasons that can explain away some of the anti-physicalist intuitions giving rise to arguments like the conceivability argument.

Methodologically, I will argue that a topic-neutral solution to the (weak) meta- problem of consciousness^4^ should be enough to naturalize a prospective theory of consciousness, provided one can show that the meta-processes and the associated phenomenal processes are not independent.

Among the possible advantages of naturalist theories of mind over their physicalist counterparts are the following:

- They are more flexible; all the theories of mind in Chalmers' A–F classification have at one time or another appeared in naturalized versions (section Chalmers's A–F Classification of Major Theories of Mind).
- They admit what I call “conciliatory” modes of explanation (section Transformational Emergence).
- They are not as metaphysically committed.
- They view science as reality's way of revealing itself to us (or to itself), cherishing instances in which empirical/synthetic discoveries trump long-held a priori intuitions (section Revelatory Dynamics).
- They are better suited to incorporate the kind of recent physics threatening to undermine physicalism (Schneider, 2017, p. 7–39);
- They are in a better position than physicalism to circumvent Kriegel's Principle of Empirical Equivalence (Kriegel, 2018, p. 15)^5^.

### 1b. Setting the Limits From Inside

As examples of Naturalism's suspicion of the ability of a priori analysis to determine the scope and limitations of the sciences (from the outside, so to speak), consider Heisenberg's uncertainty relations and Gödel's incompleteness theorem. The first results from the maturation of classical mechanics to the point of establishing some of its own scope and limitations by discovering quantum mechanical indeterminacy (Plotnitsky, 1994). The second results from the maturation of arithmetic to the point of establishing some of its scope and limitations by discovering Gödelian undecidability^6^. Both cases are examples of mature sciences establishing their own scope and limitations “from the inside,” so to speak, despite external philosophical intervention lacking in patience and underestimating the poietic capacity of the sciences. The same kind of philosophical overreach in the form of categorical Russellian Monism is the attempt to convince us that physics is not rich enough to establish the existence of intrinsic/non-relational/non-structural elements in its midst. This is reminiscent of Wolfgang Pauli's a priori comparison of the question of whether the electron is there when no one is looking to asking how many angels can sit on the head of a pin. As Mermin (1985) has shown, unlike the second question, the first led to the Aspect experiment and empirical investigations that are crucial to understanding QM. Another illuminating example of philosophical overreach is George Boole's “conditions of possible experience,” a set of a priori derived inequalities that according to Boole had to be satisfied by any real possible experience. It was Pitowsky (1994), using the famous Bell inequalities, who showed that Boole's inequalities were violated by physical results that Boole had declared a priori impossible.

### 1c. Neo-Naturalism

The “neo” in Neo-Naturalism is used in different ways and can refer to a reconfiguration of naturalism that accounts for scientific facts unknown to the naturalists of old. Examples would be quantum mechanical facts like entanglement and a more holistic naturalism such as Bohm's Implicate Order, the reliance of Ladyman and Ross's “naturalized metaphysics” on the vague ontological nature of the constituents of reality as portrayed by modern physics (the goal of “Everything must go” (Ladyman et al., 2007) of establishing a radical verificationist structural realism; symmetry principles and modern approaches to theoretical unification (Kitcher, 1981) and ontological unification based on Noether's Theorem (Neuenschwander, 2011), the construction of spacetime from primitive ultimates, the role of information in QM and General Relativity, and its implications for concreteness and causal closure (Greene, 2005).

The “Neo” can also refer to responses to naturalism that reject the uncritical reliance of naturalism on traditional metaphysical dogma. Here one can think of McDowell's “unquestioned” second nature (McDowell, 1996) as a naturalist rejection/reconfiguration of traditional naturalism or even Dewey's pragmatic naturalism, attempting to replace problematic metaphysical concepts with a naturalistic reconstruction of semantics that appeals to embedded problem solving and basic communication while preserving the principle of continuity central to Godfrey- Smith's theoretic matrix. PGS, himself a leading naturalist who relies on new empirical findings to illuminate our origins, and inspired perhaps by the pre-Socratic dictum that “The first to appear is the last to be revealed,” justifies his diachronic approach to the emergence of minds in the very beginning of his book *Other Minds: The Octopus, the Sea, and the Deep Origins of Consciousness* (Godfrey-Smith, 2016) by quoting James (1890):

The demand for continuity has, over large tracts of science, proved itself to possess true prophetic powers. We ought therefore ourselves sincerely to try every possible mode of conceiving the dawn of consciousness so that it may not appear equivalent to the irruption into the universe of a new nature, non-existent until then.

On this account PGS is a pragmatic Neo-Naturalist who believes that understanding metabolic “molecular storms” and Reafference Compensation^7^ in nematodes or zooplankton is essential to shedding light on human subjectivity. There is also the attempt of Hutto's neo-naturalism to balance traditionalist unificationist naturalism with naturalized liberal pluralism by deploying a version of naturalism associated with Wittgenstein (Hutto, 2018).

In this paper I will try to construct a neo-naturalist theory of mind that combines PGS's continuity with, among other things, Ladyman and Ross's Principle of Naturalist Closure (PNC) (p. 10, 37), necessarily relating new useful metaphysical hypothesis to fundamental physics and demanding a maximal overlap of physics with metaphysics, and Melnyk's emphasis on establishing cross-theoretical a posteriori identities (Melnyk, 2013, p. 83). To that I will add (Humphreys, 2016b) Transformational Emergence to help naturalism balance the opposing demands of continuity and novelty while keeping an eye on the meta-problem of consciousness for empirical reasons.

The neo-naturalism that I will promote is:

- Neo-Nominalist—Committed to spatiotemporal existence except when accounting for emergent spacetime.
- “Pre-Socratic”—Physics as immanent poietic revealing.
- Empirical—Deeply suspicious of a priori analytic attempts to limit the sciences (Quine).
- Patient—Takes a diachronic approach to the sciences, holding that sufficiently mature sciences determine their scope and limitations from the inside, so to speak.
- Conciliatory—Embraces the unexplained and even the unexplainable when shown to “carve nature at the joints” (Lewis, 1983; Sider, 2011; Goff, 2017a), thus enabling access to novel conceptual spaces.
- Strategic—Separates the problem of consciousness into the scientific problem of providing a full physical explanation, a formidable project indeed, and the meta- philosophical one of letting physics explain our anti-physicalist intuitions.

## Sec. 2. Chalmers' A–F Taxonomy and The Meta-Problem of Consciousness 2a

### 2a. The Meta-Problem of Consciousness

According to Chalmers (2018, p. 1), “the meta-problem of consciousness is (to a first approximation) the problem of explaining why we think that there is a problem of consciousness.” Like Chalmers, we will search for topic-neutral explanations of the behavior associated with problem reports of consciousness.^8^ In “Beyond the Neural Correlates of Consciousness,” Kriegel (2018) advances his “Principle of Empirical Equivalence,” arguing convincingly that the likelihood of discovering empirical evidence favoring one of the theories of mind in Chalmers' A–F classification of major theories of mind over the others is slim to none. However, the truth of Kriegel's conjecture does not rule out the discovery of empirical evidence that could solve the theory-neutral “meta-problem of consciousness” (Chalmers, 2018). Since naturalism itself is theory-neutral (all these theories reject the supernatural) and empirical, it makes sense to look for both realist and eliminativist naturalization strategies that concentrate on solving the meta-problem of consciousness.

Importantly, the meta-problem is not just a problem for illusionists. It is a problem for everybody. The problem of explaining our judgments about consciousness arises if consciousness is an illusion, and it also arises if consciousness is perfectly real. Furthermore, even a non-illusionist can reasonably hope both that there will be a solution to the meta-problem and that this solution will help us with the hard problem (Chalmers, 2018, p. 3).

Neo-naturalism is not as much anti-theoretical as meta-theoretical, deeply suspicious of the immutability of philosophical questions and instead asks the same questions but as products of beings like us in a world like ours (as a form of behavior) and asking how they came to be in the first place, in the best tradition not only of Dewey and Peter Godfrey-Smith but also of work on AI that generate problem reports. It is interesting to note that the theme of “questioning the question” brings together the meta-problem of consciousness, naturalism, and the postmodern critique of traditional metaphysics based on the “unquestioned privilege of the question” (Derrida in “Of Spirit,”^9^ Derrida, 1989) in the history of Western metaphysics. Both Neo-Naturalism and postmodern thinking are meta-theories that attempt to distance themselves from traditional metaphysics, converging into a space where thinkers like Derrida, Dewey, and McDowell encounter Chalmers, who in the best naturalist tradition does not discard traditional metaphysics but accords equal weight to the meta-problem and the hard problem of consciousness. The weak meta-problem embraced here presents us with other advantages:

Although the meta-problem is strictly speaking an easy problem, it is closely tied to the hard problem. We can reasonably hope that a solution to the meta-problem will shed significant light on the hard problem. A particularly strong line holds that a solution to the meta-problem will solve or dissolve the hard problem. A weaker line holds that it will not remove the hard problem, but it will constrain the form of a solution (Chalmers, 2018, p. 2).

While it is interesting to consider the prospects of the naturalization of all the theories in Chalmers's classification, A–F, of major theories of mind (2010) in conjunction with their prospects of solving the meta-problem of consciousness, I will only do so for a version of type-C theories that I consider a preferable naturalization candidate and briefly present the alternative theories.^10^

### 2b. Chalmers's A–F Classification of Major Theories of Mind

- Type-A Materialism. Eliminative Materialism and Illusionism that reject the existence of both ontological and epistemological explanatory gaps (holding both that consciousness is not made up of something non-physical and that there is no explanatory gap separating conceptual and phenomenal concepts) and considered by Dennett and Frankish, respectively, as the only viable naturalistic theory of consciousness, can be linked to Quine's “replacement naturalism” aiming to replace traditional epistemology with empirical psychology, Gilbert Ryle's scientifically austere behaviorism, and Dawking's emphasis on “natural” evolution, all respectable naturalistic strategies. When it comes to the meta-problem, both would probably point to the fact that by producing a topic-neutral solution to the meta-problem, the eliminativist has nothing more to explain, while consciousness realists must still explain the relationship between the meta-process and the more problematic phenomenal process, because if the meta-process is enough to explain behavioral/physical problem reports^11^ without consciousness, then problem reports of consciousness that are not caused by consciousness are unjustified^12^. However, consciousness realists can claim that the meta-process (the mechanism or process associated with the solution to the meta-problem) and the phenomenal process (the mechanism or process associated with the solution to the hard problem) are two aspects of the same mechanism, such that the meta-process creates conditions that make possible the instantiation of the phenomenal process constraining the hard problem. The problem with eliminativist solutions is that they are incredibly “unnatural” (Strawson, 2016) and make the kind of extreme metaphysical commitments abhorred by naturalism.
- Type-B Materialism. Papineau (2016) has sketched ways in which type-B non-reductive materialism, rejecting the existence of an ontological gap but acceping an irreducible epistemic conceptual gap, can be naturalized, and the popular “phenomenal concept strategy” is probably considered by type-B theory proponents a powerful topic-neutral solution to the meta-problem. The problem with this realist approach is that it makes the meta-process and the phenomenal process incommensurate. Also, the non-reductive commitment is unsatisfying and precisely the kind of analytic metaphysical commitment that seeks to limit the scope of the sciences, and is thus rejected by naturalism on these grounds alone.
- Type-C Materialism. Of these six types of theories of mind, only one, type-C materialism, attributes Levine's Explanatory Gap to current ignorance of experience-relevant physical facts, the other five depending on a priori analytic attempts to determine the scope and limitations of physical novelty from without, so to speak. This makes type-C theories ideally suited to benefit from empirical discoveries and naturalization. However, naturalizing type-C theories faces two major obstacles; they are notoriously unstable (see SDA^13^), and, according to Daniel Stoljar, most philosophers are reluctant to embrace theories that rely on ignorance (Stoljar, 2009). Section Two problems facing naturalized type-C theories, SDA, Stoljar's Epistemic View, and Transformational Emergence will attempt to naturalize type-C materialism by overcoming these problems. I will try to do so by embracing a weak type-C theory in which Mary, the omniscient color-blind scientist who knows all the physical facts relevant to color vision, knows why she cannot experience colors before leaving her black and white room, perhaps by discovering a relevant limiting theorem.
- Type-D Dualism. In his 1966 “The Conscious Mind,” Chalmers advances a form of Naturalistic Dualism, naturalistic because he believes mental states are caused by physical systems like brains and are related to them nomically through psycho-physical laws and dualist because he believes mental states are not reducible to physical ones. However, not only is Naturalistic Dualism ill-suited to handle the meta-problem, it also makes prohibitive metaphysical commitments, contradicting both the spirit and letter of naturalism.
- Type-E Interactive Dualism is hard to naturalize (Papineau, 2016, p. 16) because of the way spatiotemporal effects are caused by something that is external to space and time, and it is hard to see how this happens unless one embraces the kind of “deus ex machina” principles antithetical to ontological naturalism. It also does not seem to help much with the meta-problem.
- Type-F theories holding that the categorical base of the microphysical properties is phenomenal or proto-phenomenal not only make unreasonably strong metaphysical commitments but also attempt to limit the sciences from “without” by holding that even a mature physics at the limit of its theoretical validity will fail to establish the existence of non-relational/intrinsic and non-structural elements in its midst. Searle's Biological Naturalism holds that it is possible to have an epistemologically objective theory of an ontologically subjective domain. When it comes to the meta-problem of consciousness, type-F theories seem to fail to explain problem reports.^14^ Chalmers suggests that the meta-process can be realized by the phenomenal process, but this approach too is burdened by unnecessary metaphysical commitments and is harder to naturalize than what I will call type-XC materialism, which brings us to the next section.

## Sec. 3. Two Problems Facing Naturalized Type-C Theories, SDA, Stoljar's Epistemic View, and Transformational Emergence

### 3a. Naturalism and the Ignorance Hypothesis

As we have stated above, the naturalization of type-C theories must overcome both Chalmers's Structure and Dynamics Argument (SDA) and Stoljar's objection. Most type-C theories are based on currently unknown physical truths, and the best-worked-out such theory is probably Daniel Stoljar's Epistemic View. Central to this view is his Ignorance Hypothesis, holding that we are ignorant of experience-relevant physical truths, knowledge of which would be enough to explain away the conceivability argument. The naturalistic theory of mind presented here embraces Stoljar's separation of the “problem of consciousness” into two separate problems, the formidable project of providing a full constitutive and causal explanation of consciousness (beginning with the Big Bang), and the philosophical problem of explaining away the anti-physicalist intuition giving rise to arguments like Chalmers' “conceivability argument.” However, a more positive conception of the Epistemic View is not easy to come by, and according to McGinn and Russell our ignorance is of facts that are quite different than any we are familiar with, which makes it difficult to find the kind of relevant historic case studies on which Stoljar's argument depends:

Stoljar is here placing strong demands on the content of our ignorance. It must be such that if only we knew the relevant non-experiential facts, this would render zombies inconceivable. However, it is not clear that any non-experiential facts could play this role. By their nature, non-experiential facts would seem to be third-personal, objective, and non-perspectival, while experiential facts are first- personal, subjective, and perspectival. It is hard to see how knowledge of the former could automatically render the absence of the latter inconceivable (Papineau, 2007).

However, this is exactly what I will attempt to do here. While on their face Stoljar's Epistemic View and type-C theories seem ripe for naturalization, the Ignorance Hypothesis is problematic nevertheless. To quote Stoljar (2013, p. 1–2), “…while the view is attractive on the surface, most philosophers of mind don't hold it. Many of them implicitly operate under the assumption—in Wittgenstein's famous phrase—that, at least for philosophical purposes, ‘all the facts…lie open before us.’”

According to Stoljar, naturalism follows Wittgenstein here by rejecting the Ignorance Hypothesis. Appealing to truths that are “off the table” in the midst of philosophizing threatens to explain too much and restricting oneself to “on the table” facts only explains too little. It is here one can appeal to Transformational Emergence (TE) (Guay and Sartenaer, 2016), which can be used to naturalize type-C Materialism in general and Stoljar's Epistemic View in particular by replacing their need to philosophize with elements that are possible but “off the table” with elements that are “impossible” but “on the table.”

### 3b. Transformational Emergence

Naturalism has always suffered from a certain tension between its commitment to the principle of continuity and the emergence of radical novelty. Transformational Emergence is a way of balancing both commitments.

If S(t1) = S1, TE consists of a dependence clause and a novelty clause, (depd) S2 is the product of a spatiotemporally continuous process going from S1 (for example causal, and possibly fully deterministic). In particular, the “realm” R to which S1 and S2 commonly belong (e.g., the physical realm) is closed, to the effect that nothing outside of R participates in S1 bringing about S2.

And yet: (novd) S2 exhibits new entities, properties, or powers that do not exist in S1, and that are furthermore forbidden to exist in S1 according to the laws {L_*i*1_} ni = 1 governing S1. Accordingly, different laws {L_*i*2_} mi = 1 govern S2.

By embracing the “impossible which is there nevertheless” (Plotnitsky, 2000) on Lacan and the sqrt (−1)]^15^, TE provides naturalization strategies that are less explanatory and more conciliatory, having to do more with “placing” and “providing room for” such impossible but “joint carving” elements within our great naturalistic system than with defusing their paradoxical nature. TE's embrace of the impossible places it between radical and benign emergence, making it ideally suited for bridging intractable explanatory gaps. Diachronic Emergence can be said to consist of smooth evolutionary stages and short revolutionary stages connecting different evolutionary epochs that, according to TE, depend on the generation of elements that are forbidden during the initial epoch (think Archaeopteryx). Among other things, TE causes a priori analysis^16^ serious problems with information compression since it is very difficult to account for every forbidden combination from the armchair. Like naturalism, TE emphasizes spatiotemporal continuity and is ideally suited to handle novelty without appeals to the extra-theoretic. In section Three Physical Examples that can Make a Philosophical Difference I will use TE as a guide for physical theories that are “strange enough” to make a philosophical difference.

### 3c. SDA and Type-XC Theories

The second problem facing type-C theories is the theoretical instability problem and the SDA (Chalmers, 2010, p. 39–40), which holds that:

- A physical description is structural/dynamic.
- Structural descriptions can only yield other structural descriptions.
- Consciousness is not structural.

Therefore, physical description, even at the limit of its theoretical validity, fails to apprehend consciousness. The SDA is especially damaging to type-C theories because of their reliance on the prospects of currently unknown physics and can be used to show (Alter, 2015) that type-C theories must collapse into either type-A, type-B, type-D, or type- F theories.

By structure/dynamic, Chalmers refers to the familiar dynamic equations of physics, where a structural description is defined as a Ramsey sentence whose O-terms are spatiotemporal, mathematical, and nomic. Stoljar (2013, pp. 21–23) argues against the SDA and Chalmers' use of “structure”; however, emergent spacetime, consisting of proto-temporal and proto-spatial elements, results in a more physical lack of structure that cannot be given a spatiotemporal description or described by the ordinary dynamic equations of physics due to lack of symplectic invariance^17^. I will have more to say about the SDA in section Self-measurement, circumventing the SDA in the context of self-measurement.

Realist solutions to the (weak) meta-problem of consciousness that constrain the hard problem are not easy to come by, and both the meta-process and the phenomenal process are likely to be strange, as parsimony suggests it is unlikely that two highly correlated strange phenomena will be explained by two unrelated strange and rare mechanisms. Therefore, a topic-neutral mechanism that explains the meta-problem but not the full-blown hard problem can nevertheless provide the “end of the string” that could unravel the hard problem. Again, if a realist is compelled to accept the validity of some meta-process while insisting it is independent of the phenomenal process, then she might as well embrace eliminativism.

To solve both the type-C instability problem and the meta-problem of consciousness, it is enough to embrace Chalmers's “Intermediate Notion Type-C Theory,” (Chalmers, 2010, pp. 121–122) a two-stage theory that can be termed Type-XC theory:

One might hold that there is some intermediate notion X, such that truths about X hold in virtue of structural-dynamic descriptions, and truths about consciousness hold in virtue of X. But as in the case of type-A materialism, either X is functionally analyzable (in the broad sense), in which case the second step fails, or X is not functionally analyzable, in which case the first step fails.

Here I think that Chalmers is too pessimistic for several reasons. First, type- XC theories seem ideally suited to relate the meta-process to the phenomenal process while avoiding unnecessary metaphysical commitments like Russellian Monism's commitment to “phenomenal stuffing” (Armstrong, 1968). Second, I think that there is no reason to claim that structural/dynamic processes cannot produce non-structural states, especially if we consider emergent spacetime. If the linear, ordered time of physics that underlies the symplectic-invariant dynamic equations of physics emerges from a non-ordered primitive proto-temporal state through symmetry breaking, then that broken symmetry can be restored. A priori assumptions holding that physics is not rich enough to establish the existence of non-structural elements in its midst are not justified (consider singularities) and constitute the kind of analytic overreach opposed by naturalism.

If type-C theories hold that Mary, the color-blind scientist, will be able to experience red when she knows enough about the physical facts, type-XC theories hold that long before that Mary will know enough to understand why she cannot experience red before exiting her room, perhaps by discovering a limiting theory. Another way of thinking of type-XC theories is as a search for a single mechanism that has a meta-process aspect and a phenomenal aspect in which a structural meta-process necessitates a non-structural phenomenal process.

## Sec. 4. Unificationist Poietic Naturalism, Symmetry Considerations, and “Pragmatic Neutral Monism”

### 4a. Unificationist Neo-Naturalism

As stated in section Naturalism and Neo-Naturalism, the version of unificationist naturalism that I have in mind combines elements of Kitcher's (1981) theoretical unification, Oppenheim and Putnam's scientific unification (Oppenheim and Putnam, 1958), Ladyman and Ross's “Everything Must Go” (Ladyman et al., 2007), and Melnyk's (2013) establishment of cross-disciplinary a posteriori identities.

A classic reference favoring naturalistic unification is Oppenheim and Putnam's “The Unity of Science as a Working Hypothesis” (Oppenheim and Putnam, 1958), in which:

Oppenheim and Putnam intended to articulate an idea of science as a reductive unity of concepts and laws to those of the most elementary elements. They also defended it as an empirical hypothesis—not an a priori ideal, project or precondition—about science. Moreover, they claimed that its evolution manifested a trend in that unified direction out of the smallest entities and lowest levels of aggregation (Cat, 2017, p. 39).

Oppenheim and Putnam's empirical approach to unificationist naturalism can be supplemented by Don Ross, James Ladyman, and David Spurrett (RLS)'s “Every Thing Must Go: Metaphysics Naturalized” (Ladyman et al., 2007), which has attractive features relevant to the neo-naturalism considered here and is considered by Melnyk (2013) to currently be the “richest account to date (i) of why mainstream analytic metaphysics is objectionably non-naturalistic and (ii) of how metaphysics might be naturalized.”

RLS's project is unificationist since they aim to “defend a radically naturalistic metaphysics. By this we mean a metaphysics that is motivated exclusively by attempts to unify hypotheses and theories that are taken seriously by contemporary science.” RLS's naturalized metaphysics is clear about its rejection of philosophical a priori attempts to limit the scope of the sciences: “…science respects no domain restrictions and will admit no epistemological rivals (such as natural theology or purely speculative metaphysics).” RLS also implicitly suggest that the SDA that so threatens type-C materialism should not be taken too seriously: “…no hypothesis that the approximately consensual current scientific picture declares to be beyond our capacity to investigate should be taken seriously.”

RLS's neo-naturalized metaphysics distances itself from the cozy relationship between traditional naturalism and metaphysics and supplements the principle of causal closure threatened by new scientific conceptions of time with its PNC or “Principle of Naturalistic Closure”:

Any new metaphysical claim that is to be taken seriously at time *t* should be motivated by, and only by, the service it would perform, if true, in showing how two or more specific scientific hypotheses, at least one of which is drawn from fundamental physics, jointly explain more than the sum of what is explained by the two hypotheses taken separately (Ladyman et al., 2007, p. 37–38).

The PNC is useful to neo-naturalist theories of mind that appeal to fundamental physics and is commensurate with RLS's commitment to a maximal overlap between naturalized metaphysics and fundamental physics. It is also unificationist and poietic. However, RLS's commitment to a purely relational base conflicts with Newman's Paradox (Goff, 2017b, p. 53) and is an example of the kind of radical analytic metaphysical commitments conflicting with the naturalistic principles advanced here. I also agree with Melnyk that RLS's extreme deprecation of philosophy is unjustified and find his “a posteriori identity version” of empirical unification indispensable to consciousness realists, who believe that identifying the physical correlates of consciousness is an essential part of any scientific investigation of consciousness, especially one that is based on fundamental physics.

If a metaphysical question can be put into the “What is ?” form (e.g., “What is causation?”), then in principle it can be answered by assembling empirical evidence for the relevant a posteriori identity claim. And the same approach can be used to address the question of how to unify the sciences, since, at least on my view, unification is achieved by discovering cross-scientific a posteriori identity claims (Melnyk, 2013, p. 93).

The advantage vantage of the PNC is that it tries to account for novelty, perhaps motivated by the belief that the path of a “joint carving” unificationist naturalism is littered with novelty.

### 4b. Revelatory Dynamics

This is a good place to sketch some of the connections between ontological and methodological unification on the one hand and the kind of poietic take on the sciences advanced by this paper. Cat on Oppenheimer and Putnam naturalism states:

In an important sense, the evolution of science recapitulates, in the reverse [my emphasis], the evolution of matter, from aggregates of elementary particles to the formation of complex organisms and species (we find a similar assumption in Weinberg's downward arrow of explanation). Unity, then, is manifested not just in mereological form, but also diachronically, genealogically or historically (Cat, 2017, p. 39).

I find this quote to be closely related to the pre-Socratic dictum, “The first to appear is the last to be revealed,” mentioned by Heidegger in *Questions Concerning Technology* (Heidegger, 1977). The pre-Socratic view of physics as simultaneously revealing and concealing is commensurate with both realism and XC-type theories based on a single mechanism that reveals the meta-processes but conceals the phenomenological processes (of course there is no reason why the reason for the concealment itself should not be revealed!). To a (structural) realist, meaningful unification carves nature at the joints (Lewis, 1983; Sider, 2011; Goff, 2017a), and one of the symptoms of a truly joint carving theory is the unintended discovery of novel and highly counter-intuitive constituents of reality underlying new conceptual spaces. Unification offers the best and perhaps the only way of advancing toward “cutting nature at the joints.”

A “pre-Socratic” neo-naturalism views science as a poietic source of novelty that is revealed by instances of unification, as can be seen from historic examples. The counter-intuitive novelty that “sprang out” of Faraday and Maxwell's unification of electricity and magnetism was the realization that light is electromagnetic waves, just as the unification of Maxwell's equations with classical mechanics (the Galilean transformation) resulted in the highly counter-intuitive special relativity, and unifying QM with special relativity resulted in the Dirac equation and the highly counterintuitive negative energy states and antimatter. The same can be said about Newton's discovery of “action at a distance” and many more such examples that support the claim that unification is poietic.

A naturalism that wishes to account for all of these instances of unificationist revelatory dynamics must acknowledge the centrality of symmetry and invariance transformations to physics, mathematics, and cognition. The single most important tool in which physicists use symmetry principles to generate physical novelty, especially novel conserved quantities, is *Noether's Theorem*.

### 4c. Noether's Theorem and Conserved Quantities

Noether's Theorem relates conservation laws to symmetries of space, time, and internal symmetries.

Because of the central role of conservation laws, Noether's Theorem may be one of the most strategic programs of deductive reasoning in all of physics. In some sense it surely takes us along the way toward the foundation of physics (Neuenschwander, 2011, p. 5).

The theorem furnishes profound connections between the fundamental constituents of reality and symmetry by combining the calculus of variations and QM with Lie groups. More importantly for our purpose, the unification program based on this theorem attributes the differences between elementary constituents of matter to the action of “broken symmetry” mechanisms, prior to which the differences were indiscernible.^18^ Electrons and neutrinos seem very different, as do photons and Z particles, but electroweak unification [conveniently captured by SU(p. 2)] shows that they are related because the present differences between them resulted from prior symmetry breaking (Livio, 2006). By the same token, the relationship between fermions and bosons and a possible “theory of everything” depends on the prospects of supersymmetry and its mysterious connections to the exceptional finite Lie group E8 (Ronan, 2006).

If consciousness is physical, we need to explain why it appears to be so different from ordinary physical constituents. A realist assuming that consciousness is composed of some strange state of matter can ask whether symmetry principles in general and Noether's Theorem in particular can help us get a handle on that difference. Failure to do so would strengthen the conclusion that even if consciousness is a fundamentally “broadly physical” constituent of reality, it is fundamentally different than the rest of the unified constituents.

Not only do symmetry principles underlie physics and mathematics, they are also deeply implicated in their mysterious and “unreasonable” connections and seem ideally suited to apply to the *different* ways in which the *single* realist's world reveals itself.

Invariance transformations and symmetry are not just central to cognition and what we call persistence conditions, but even to modern attempts to naturalize phenomenology (Varela et al., 1991). Husserl described his Eidetic phenomenological reduction as:

…a Form of Imaginative Variation by Which You Attempt to Reduce a Phenomenon into its Necessary Essences. This is Done by Theoretically Changing Different Elements (While Mentally Observing Whether or not the Phenomenon Changes) of a Practical Object to Learn Which Characteristics are Necessary for it to be it Without Being Something else? (Wikipedia contributors, 2018).

Noether's Theorem provides a neutral monism (NM) with a twist. The four standard varieties of NM are ones where the base is phenomenal, physical, both, or neither (Stubenberg, 2018). However, if it can be established that the laws of physics are invariant under any linear combination of the physical and phenomenal parts of the base, then we may get a continuous symmetry transformation suggesting an associated conserved quantity.

The philosophical framework best suited to benefit from modern conceptions of symmetry is “common-origin neutral,” and in the next section I will present such a framework, relate it to (weak) type-C theories, and consider information as the neutral base.

## Sec. 5. (Neo)-Naturalizing Information and Pragmatic Neutral Monism

### 5a. Common-Origin Pragmatic Neutral Monism

Detractors of NM charge that it harbors two explanatory gaps, that of constructing the phenomenal from something that is not phenomenal (the protophenomenal gap) and that of constructing the physical from something that is not physical (the protophysical gap), and is therefore worse off than both physicalism and panpsychism. However, this charge fails when the protophenomenal gap is deeper than the protophysical gap, because bridging the protophysical gap improves the prospects of bridging the protophenomenal gap by identifying the base. This suggests an approach that I term Pragmatic NM (PNM) to bridging the protophenomenal gap that first looks to physics for possible base candidates to be subsequently evaluated for protophenomenal properties. One way to combine PNM with XC-type theories is to associate the first structural stage X with the restoration of a broken temporal symmetry, and the second stage, in which truths about consciousness hold in virtue of X, with the breaking of such symmetry. As stated above, one can think of X in XC-type theories as a mechanism that has two aspects, a structural one that is enough to solve the meta problem and a non-structural aspect, perhaps an inaccessible “radical interiority” (Plotnitsky, 2002), composed of physically realized information, that is not accessible to ordinary measuring devices. What makes such XC-type theories different from dual aspect theories of mind is that the non-structural phenomenal aspect of X is necessitated by its structural aspect, perhaps in the same way that the non-structural inner properties of a physical singularity are necessitated by its structural outer description (you cannot place a physical measuring device at the center of a black hole or a parallel universe).

Another way to combine PNM with XC-type theories is to identify the neutral base with the intermediate notion X so that truths about X are discovered by physics and hold in virtue of structural-dynamic descriptions, while truths about consciousness hold in virtue of X; however, the challenge is still to establish some kind of necessary connection between the two sets of truths. PNM's solution to the meta-problem of consciousness is that despite the lack of an ontological gap, consciousness cannot be grounded in ordinary physical constituents unless these are cashed back into the neutral base by undergoing symmetry restoration; presumably such mechanisms are special enough to account for our anti-physicalist intuition but not too special to be instantiated in physical systems like brains.

It seems as though a naturalized neutral monism that takes modern science seriously must consider information a serious base candidate.

### 5b. Neo-Naturalizing Information

Another way in which modern physics pressures naturalism into a radical reconfiguration worthy of the prefix *neo* is by forcing it to reconsider its nominalism on information. To an ontological naturalist, information is *abstract*, lacking causal powers above and beyond those of its physical realizers; however, modern physics strongly suggests that information is not simply abstract, supporting theories that attempt to ground concrete consciousness in information. (I will give some supporting examples from QM and GR.) In Section Naturalism and Neo-Naturalism we mentioned the centrality of spatiotemporal causal closure to ontological naturalism, and here I suggest that modern physics, especially QM, motivates replacing spatiotemporal causal closure with information-based causal closure. Hopefully the encounter between the physics of spacetime and the physics of information will be useful (PNC).

Underlying both unificationist naturalism and information-based spacetime is the concept of “difference.” As stated above, in modern physics the way to apprehend the difference between two physical constituents like a fermion and a boson is to discover a symmetry operation that transforms the one into the other (Livio, 2006); the difference is attributed to the symmetry breaking undergone by their common origin. If (space) time emerges from entangled pre-geometric q-bits, then neo-ontological naturalism may have to broaden its embrace of spatiotemporal causal closure to include information-based causal closure (causality in physics forbids faster-than-light information transfer). If deep connections exist between information and time, then those may very well be relevant to the “physics of consciousness.” The problem with information-based theories of consciousness is that information is not concrete while consciousness is. Ontological naturalism's commitment to causal closure forces it to reject any physical effects that information might have beyond the causal effects of its physical realizers; however, things change when information is used to construct spacetime and concreteness loses its meaning. While this is not the place to review the fundamental role that information plays in both QM and general relativity, it is interesting to note that while the “physical” QM wave function displays faster-than-light behavior, it is “abstract” information that is slower than light and is therefore more “real.” For an event in point A to cause an event, or a measurable change, in point B, information must be transferred between the two points. It was Asher Peres (1990) that showed that faster-than-light communication would allow perpetual motion devices and violate the no-cloning theorem. Recent work on the foundations of QM relates “causal information” to non-analytic regions in the temporal evolution of the wave function that cannot exceed the speed of light (Garrison et al., 1998; Wynne, 2002). As a matter of fact, even lack of information can have physical consequences, as in the case of ignorance of “which way information” (Hofmann et al., 2002) causing entanglement between distant rubidium atoms. General Relativity also suggests that information is not merely abstract; consider the Bekenstein Bound (Bekenstein, 2007) and the fact that the self-collapse of matter into a singularity depends exclusively on the bit number per unit area and not on the physical realization of the bits. This is the closest we get to causally efficacious information independent of its physical realizers. The Bekenstein Bound also means that information is intrinsic and Lorenz invariant.

Bits are abstract differences, and using these abstract differences to construct time may seem unnatural; abstract differences just seem too impoverished to yield time and phenomenality. However, one can argue that “difference” is more fundamental than time, because while there is no good reason to rule out possible worlds with time^*^ that functions like time yet differs from our time, it is hard to conceive of a possible world with a difference^*^ that functions like difference yet is *different* (^*^?) from our difference.

## Sec. 6. Three Physical Examples That Can Make a Philosophical Difference

I will end by using both TE and the Parsimony Conjecture to motivate the consideration of three possible modern physical mechanisms based on self-measurement, prototemporal symmetry breaking, and Maldacena's holographic principle (Maldacena, 1998) that can solve the meta-problem, constrain the hard problem, and underlie the kind of realist neo-naturalist theories of mind considered here.

In section Two problems Facing Naturalized Type-C Theories, SDA, Stoljar's Epistemic View, and Transformational Emergence we mentioned the Parsimony Conjecture available to consciousness realists and relevant to the meta-problem. Two properties of consciousness feeding anti-physicalist intuitions are its inaccessibility to physical measuring devices and its peculiar and unmediated self-access. Both properties are not only very strange but highly correlated and therefore likely to result from a single mechanism, since otherwise we would have two highly correlated strange properties that are generated by completely different strange mechanisms. This suggests a naturalist strategy that attempts to bridge the protophenomenal gap by discovering physical mechanisms that satisfy the parsimony conjecture on the one hand and that can be shown to constitute a physical correlate of consciousness on the other.

### 6a. Self-Measurement, Circumventing the SDA

As stated in section SDA and Type-XC Theories, type-C theories' appeal to the richness of physics makes them especially susceptible to Chalmers's SDA, which holds that since physical description is structural/dynamic, and since structural/dynamic descriptions can only produce other such descriptions, physics is not rich enough to describe consciousness since it is not structural. The naturalized type-C theory that I am considering here circumvents the SDA in different ways. The SDA takes structural descriptions to be spatiotemporal and mathematical, while the theories considered here are neither spatiotemporal nor symplectic, as, after all, the restoration of broken symmetry results in a lack of structure. However, ontological naturalism relies on physical measuring devices, and the argument against type-C theories can be recast thus:

- Something is physical if it can register on a physical measuring device expressible in mks units (meter, kilogram, second).
- Consciousness is not measurable by measuring devices nor expressible in mks.
- Conclusion: consciousness is not physical.

One way out for a physicalist is to note that the brain is a physical device that measures/registers instances of consciousness, and thereby conclude that consciousness is physical after all. If the brain makes measurements on itself, there are three possibilities: either the whole brain measures itself, or some subsystem A performs measurement on another subsystem B, or else some subsystem A performs measurement on itself. If A measures B, then external devices could measure B. Since it is unlikely that the whole brain measures itself, what is left is A measuring itself and so on. At some point we need a genuine intrinsically reflexive self-measurement^19^ where the measuring device is also that which is being measured and which therefore harbors physically realized information that is not accessible to any other system. One can explore self-measurement by considering an energy-conserving closed-system QM measurement like Steiner and Randel's Internon theory (Steiner and Rendell, 2018). Such theories provide a topic-neutral solution to the meta-problem by explaining why consciousness is inaccessible to external measuring devices, and are commensurate with the parsimony hypothesis and well-suited to be cast as type-XC theories. One can think of the meta-process and the phenomenal process as two aspects of the same mechanism, in which a structural aspect establishes the existence of a “non-structural” aspect where, *unlike* ordinary dual-aspect theories, the two processes are dependent.

While the first example was meant as a neo-naturalist theory of mind that circumvents the SDA, the next two examples appeal to modern physical conceptions of spacetime.

Neo-naturalist theories of mind in which time is constructed from aggregates of entangled, pre-geometric, and prototemporal^20^ bits obscure the difference between unificationist type-C theories and protophenomenal theories like “common-origin neutral monism” in which the mental and the physical share a common origin. Perhaps this is a sign of progress. By the time we dig this deep, our traditional ontological notions are so far removed from what we are examining that any base that can be used to construct both the mental and the physical is welcome^21^. In the next example, the differences between XC-type theories and PNM are insignificant.

### 6b. Chronos, Kiros, and Cutting Space in Two

Considering an emergent time, we can define prototemporal properties similarly to the way Chalmers defines “protophenomenal” properties such that a prototemporal property:

- Is not temporal.
- Is non-structural.
- Necessitates the temporal in the right context.

One way to bridge the prototemporal gap is by having the prototemporal phase/state undergo symmetry breaking, producing our familiar ordered, linear, and chronological Lorenzian time. The ancient Greeks called this time “Chronos,” but they had another word for the experience of the passage of time, Kiros.^22^ It is possible that the prototemporal state can also undergo symmetry breaking that, unlike the flat open-ended Chronos, results in a reflexive state, similar to a closed timelike curve (CTC) or a time loop.^23^ The question here is not whether this possibility is ‘too strange’, but whether it is strange enough to make a philosophical difference and not so strange as to be ruled out theoretically; it may very well be the kind of forbidden property crucial to successful transformational emergence. CTCs were discovered by Gödel (1949) as legitimate solutions to Einstein's gravitational equations, and have been considered non-physical because of grandfather-type paradoxes. Max Tegmark attempted to prove that CTCs cannot harbor physical bits, but Seth Lloyd (Lloyd, 2011) showed that they can harbor qbits. Aaronson (2005) suspects that they are not physical because that could allow the construction of hyper-computers that can solve NP-complete problems.^24^ At the same time, new spatiotemporal formulations of QM are based on ER = EPR (Maldacena and Susskind, 2013) or the equivalence of the Einstein-Rosen bridge (a wormhole connecting two black holes) and quantum mechanical entanglement. One way or another, it is fair to say that while the ontological status of CTCs is controversial, they are the kind of TE transgressions that can open up new important conceptual spaces. If you are a super-substantivalist^25^ who believes in the reality of the continuum, then projective geometry endows CTCs with a one-to-one mapping between the infinite time-line and the temporal unit circle, creating an interesting connection between the transcendent infinite Chronos and the immanently reflexive Kiros. To see how CTCs satisfy the parsimony hypothesis to a tee, one can consider an ancient philosophical question: Can space be cut in two? (Callender and Weingard, 2000).

From Descartes to Kant to Quinton (1962) and Smart (1964, p. 1–23) philosophers have struggled with this problem but were ill-equipped to handle it. The 3-dimensional problem was only solved in a 4-dimensional framework as a case of time-dependent spatial topology in 1967 by the topologist Geroch (1967), who proved that it is possible to cut space in two, but there is a temporal price to be paid^26^ because the resulting space must either be temporally non-orientable or else exist in (as) a closed time-like curve. This is a beautiful example of the way in which modern notions of spacetime provide us with philosophically meaningful novel moves. Not only do the spatial demarcation conditions of such systems make their information inaccessible to external devices, but they also constrain their internal temporal architecture in a way that causes this information to acquire *sui generis* self-access, satisfying David Deutsch's (Deutsch, 1991) consistency conditions and enabling it to survive the grandfather paradox. The added benefit of this approach is that it portrays the “other minds problem” and the radical spatial discontinuity of consciousness on the one hand and its peculiar unity, self-givenness, and unmediated intimacy on the other as two sides of the same coin. Showing that such mechanisms also constitute a minimal physical correlate of consciousness would be enough to explain why zombies are highly unlikely but not a priori impossible. This is an empirical topic-neutral approach suggesting that the reason it is hard to disprove the conceivability argument is because we have a physical system, similar to an island universe (Lewis), into which it is objectively impossible to insert measuring devices. Such mechanisms satisfy the parsimony hypothesis, and qbits in a CTC may possess *sui generis* continuous symmetries to which Noether's Theorem can be applied and that are ideally suited to harbor a “view from the inside.”

On this analysis, the hard problem is hard because explaining consciousness requires more than explaining behavioral functions. Even after we have explained all the behavioral functions that we like, there may still remain a further question: why is all this functioning accompanied by conscious experience? When a system is set up to perform those functions, from the objective point of view, why is there something it is like to be the system, from the subjective point of view? (Chalmers, 2018, p. 1).

Again we have an example of a two-stage XC-Materialism or a mechanism with a structural aspect that is enough to serve as a meta-process (explaining problem reports) but that also is implied to harbor a non/structural aspect that could both serve as X in the XC and the phenomenal process necessitated by the meta-process.

### 6c. Holographic Possibility

The third and final example of the way in which the “new physics” can make a philosophical difference involves the *holographic principle*. In his *The Fabric of the Cosmos* (Greene, 2005, p. 487–8), Brian Greene, who believes that future physical theories will be background-independent, thinks that they are likely to emerge from a unifying background-dependent string theory with background-independent quantum loop gravity (Greene, 2005, p. 490), and one of the reasons he believes that spacetime is not primitive is Maldacena's “holographic principle,” which establishes an equivalence between n-dimensional conformal quantum field theories without gravity and n+1 dimensional theories with gravity in an Anti-de-Sitter space (CFT-ADS duality). The holographic principle has already been used in condensed matter theory by Sachdev (2013) to solve intractable two-dimensional problems like the Quantum Nernst effect by switching to the dual three-dimensional problem. The ontological significance of the holographic principle is still not clear, especially since there are good reasons to believe that our own universe is really a two-dimensional surface containing two-dimensional sub-systems that generate three-dimensional illusions, or better, systems that access the three-dimensional view dual to the surface. To quote Brian Greene from *The Fabric of the Cosmos,*

After including additional curled-up dimensions as required by string theory, Maldacena convincingly argued that the physics witnessed by an observer living within this universe (an observer in the soup) could be completely described in terms of physics taking place on the universe's boundary (physics on the surface of the soup can) (Greene, 2005, pp. 483–484).

Greene goes on to add that (Greene, 2005, p. 485): “…this provides yet another hint that spacetime is not fundamental. Not only can the size and shape of spacetime change in the translation from one formulation of a theory to another, equivalent form, but the number of space dimensions can change, too.” However, in our “observable” three-dimensional universe we discover brains in which we proceed to look for the neural correlates of consciousness (NCC includes physical correlates in general). One of the better current NCC candidates is the Claustrum (Reardon, 2017), which happens to be a thin dense information hub of a membrane with a quasi-conformal structure. If one could show that the Claustrum contains a large number of entangled electrons describable by a two-dimensional conformal field theory, it would have a dual three-dimensional system internal to the surface with an Anti-de-Sitter geometry (containing a singularity), thereby establishing a strange theoretical connection between two-dimensional quantum criticality and three- dimensional black hole dynamics (Sachdev, 2013).

All this is related to the canonical question regarding the fate of information inside black holes. For example, Hawking et al. (2016) in his latest theory suggested that the information is not destroyed but ends up in a very exotic state resembling a holographic halo. Such a holographic scenario would be compatible with a yin-yang view in which the two dual systems harbor each other. Here a solution to the meta-problem would note that when you look at a Claustrum from without, you see a two-dimensional surface instantiating one kind of physics, and when you are within a Claustrum you get the dual three-dimensional view internal to the surface, instantiating another physics. Discovering such a CFT process in the Claustrum would provide a meta-process (or X in XC-type theory) and a related, poorly understood phenomenal process.

After centuries of thought we still can only portray space and time as the most familiar of strangers. They unabashedly wend their way through our lives, but adroitly conceal their fundamental makeup from the very perceptions they so fully inform and influence (Greene, 2005, p. 492).

Nothing has proven as contrary to common sense as space and time, and to quote Greene again:

Over the last century, we've become intimately acquainted with some previously hidden features of space and time through Einstein's two theories of relativity and through quantum mechanics. The slowing of time, the relativity of simultaneity, alternative slicing of spacetime, gravity as the warping and bending of space and time, the probabilistic nature of reality and long range quantum entanglement were not on the list of things that even the best of 19th century physicists would have expected to find just around the corner. And yet there they were (Greene, 2005, p. 492).

Does spacetime have more surprises in store? Can those surprises be strange enough to shed some light on the physics of consciousness? Here I have tried to explore some alternatives that were strange enough to make a philosophical difference but not so strange as to be unphysical. While all this may sound a bit far out, the point I wish to make is that a neo-naturalism that trades its spatiotemporal causal closure for an information-based approach and that attempts to account for novel scientific conceptions of spacetime has new philosophically relevant moves at its disposal, and that the crisis of ontological naturalism presents us with fascinating opportunities.

## Author Contributions

The author confirms being the sole contributor of this work and has approved it for publication.

### Conflict of Interest Statement

The author declares that the research was conducted in the absence of any commercial or financial relationships that could be construed as a potential conflict of interest.

## Footnotes

^3^Theories that do not unfold in preexisting spacetime.

^4^Think of designing AI that evolves to the point where it claims to be conscious or embraces dualism while lacking consciousness.

^5^Because concentrating on the meta-problem of consciousness can preserve both their neutrality and their reliance on empirical input.

^6^Gödel achieved this breakthrough by managing to have numbers refer to themselves.

^7^An unintended consequence of the need of embedded systems with a low signal-to-noise ratio to filter out the noise generated by the systems themselves. To do so, biological systems evolved self-models, thus providing a topic-neutral explanation of primitive subjectivity. The “first to appear” proto-subject is the “last to be revealed” to the subject's search for understanding. This is reminiscent of the pre-Socratic “ascent by descent” but also of Putnam's “Unification in reverse.” More on that in section Unification, Poiesis, and Symmetry.

^8^A typical topic-neutral explanation would attempt to show that computers with the right architecture, say a self-referential one, are likely to display symptoms typical of those caused by the meta-problem *without any consciousness!*

^9^In Derrida's critique of Heidegger's logocentric claim that “questioning is the piety of thought.”

^10^Another legitimate strategy attempting to provide a topic-neutral solution to the meta-problem of consciousness that does not fit well in Chalmers's A–F spectrum is Uriah Kriegel's self-representational theory of consciousness, which attributes our illusion of the non-functionalizability of consciousness to resonant firing interactions between the dorsolateral prefrontal cortex and the sensory cortex (McClelland, 2016).

^11^Think of a solution to the meta-problem as showing that evolving embedded robots devoid of consciousness are eventually compelled to make such reports, say, because of evolving self- representational software.

^12^At most a Gettier case of true but unjustified belief.

^13^Chalmers' Structure and Dynamics argument; see section The SDA and Type-XC Theories.

^14^Even if one tries to overcome charges of epiphenomenalism by holding that the microphysical properties are properties of their phenomenal or proto-phenomenal categorical base, the problem of how reports about consciousness are actually caused by the categorical base above and beyond its relational dispositions is not clear.

^15^The sqrt(−1) is a forbidden combination of sqrt and −1 that made possible complex analysis, including important aspects of quantum mechanics, and provided access to vast novel conceptual spaces. However, TE is also relevant to Gould's “punctuated equilibrium” and the crucial dependence of 1st order phase transitions on statistically rare intermediate structures.

^16^True for an ideal reasoner but not for Laplace's demon.

^17^Symplectic invariance refers to the invariance of the equations of motion to spatio-temporal translations. Conservation of energy and momentum depend on the homogeneity of time and space, respectively. Strong fluctuations in the spatiotemporal manifold make it impossible to conserve the structure of a system's equations of motion during its evolution. See Canonical Invariance (Landau and Lifshitz, 1975).

^18^For Leibniz, symmetry is a way in which indiscernible differences illuminate discernible ones.

^19^For example, see David Albert's *Quantum Mechanics and Experience* (Albert, 1994).

^20^I define “prototemporal” similarly to Chalmers' definition of “protophenomenal.” A proto(spatio)temporal property is one that is not (spatio)temporal or structural and is such that it necessarily yields (spatio)temporality when arranged properly.

^21^(This is *not* a case of a type-C theory collapsing into a type-F theory.

^22^Emanuel Levinas said that “consciousness of time is the time of consciousness.” (Drabinski, 2001, p. 146).

^23^Perhaps in the same way that photosynthesis is an example of biomimesis where biological systems reach a theoretical physical limit (energy conversion efficiency), brains reach some information-theoretic limit resulting in the formation of physical singularities and CTCs.

^24^Because if you can solve one such problem you can solve all such problems.

^25^Holding that spacetime is substantive and that everything is constructed from it (topological particles).

^26^Compare that to more elaborate models of cutting space in two, as in Ramssdonk (2010).
